# Contextual factors affecting hint utility

**DOI:** 10.1186/s40594-018-0107-6

**Published:** 2018-04-14

**Authors:** Paul Salvador Inventado, Peter Scupelli, Korinn Ostrow, Neil Heffernan, Jaclyn Ocumpaugh, Victoria Almeda, Stefan Slater

**Affiliations:** 1 School of Design, Carnegie Mellon University, 5000 Forbes Avenue, Pittsburgh, PA USA; 2 Worcester Polytechnic Institute, 100 Institute Road, Worcester, MA USA; 3 University of Pennsylvania, Philadelphia, PA USA; 4 Teachers College, Columbia University, 525 W. 120th Street, New York, NY USA

**Keywords:** Hints, Context, Experimental methodology, ASSISTments

## Abstract

**Background:**

Interactive learning environments often provide help strategies to facilitate learning. Hints, for example, help students recall relevant concepts, identify mistakes, and make inferences. However, several studies have shown cases of ineffective help use. Findings from an initial study on the availability of hints in a mathematics problem-solving activity showed that early access to on-demand hints were linked to lack of performance improvements and longer completion times in students answering problems for summer work. The same experimental methodology was used in the present work with a different student sample population collected during the academic year to check for generalizability.

**Results:**

Results from the academic year study showed that early access to on-demand-hints in an online mathematics assignment significantly improved student performance compared to students with later access to hints, which was not observed in the summer study. There were no differences in assignment completion time between conditions, which had been observed in the summer study and has been attributed to engagement in off-task activities. Although the summer and academic year studies were internally valid, there were significantly more students in the academic year study who did not complete their assignment. The sample populations differed significantly by student characteristics and external factors, possibly contributing to differences in the findings. Notable contextual factors that differed included prior knowledge, grade level, and assignment deadlines.

**Conclusions:**

Contextual differences influence hint effectiveness. This work found varying results when the same experimental methodology was conducted on two separate sample populations engaged in different learning settings. Further work is needed, however, to better understand how on-demand hints generalize to other learning contexts. Despite its limitations, the study shows how randomized controlled trials can be used to better understand the effectiveness of instructional designs applied in online learning systems that cater to thousands of learners across diverse student populations. We hope to encourage additional research that will validate the effectiveness of instructional designs in different learning contexts, paving the way for the development of robust and generalizable designs.

## Background

In the context of the STEM Grand Challenge run by the Office of Naval Research, this work aims to further the state-of-the-art in intelligent tutoring systems not only by describing the infrastructure developed by Worcester Polytechnic Institute (WPI) to run experiments but also report on one such study. Aimed toward improving learning in such interactive environments, it is important to consider the types of help and feedback that are most effective to benefit learners. It is also imperative to understand how the context of the learning environment influences the efficacy of such pedagogical techniques.

Several studies have shown that help strategies, such as on-demand hints, lead to better learning outcomes (Aleven et al. 2006; Campuzano et al. 2009; VanLehn 2011). Unfortunately, it is often the students who are most in need of assistance that fail to seek help (Puustinen 1998; Ryan et al. 1998), and those who seek it may not use what is given effectively. Aberrant help use may actually decrease learning. For example, students may use on-demand hints to retrieve the correct answer without understanding the reasoning behind their response (Aleven and Koedinger 2000), engage in systematic guessing to identify the correct answer (Baker et al. 2004), or simply ignore available help altogether (Aleven et al. 2003). A recent study (Inventado et al. 2016) examined performance differences among students who had varied access to hint content while answering problem sets in ASSISTments, an online learning platform, to solve mathematics problems as part of their summer preparatory activities. Such summer activities usually consist of problems, assigned either by the student’s incoming teacher or by a district-level administrator, which students are urged to complete by the start of the new school year. Students are typically assigned these activities at the beginning of the summer and are typically graded (for completion) at the start of the next school year. It should be noted that schools do not uniformly assign summer activities, and that expectations may differ across grade and course levels. The practice is typically meant to keep students thinking about math throughout the summer months as an attempt to deter skill regression. Packets may be assigned to all students uniformly or to those in need of extra assistance or attention. Within the randomized controlled experiment conducted by Inventado et al. (2016), the treatment group was able to request a series of progressive hints, while the control group was only able to access a bottom-out hint (one which provides the answer). Although these conditions were applied only to the first three problems—after which, both groups were able to access several hints per problem—Inventado et al. (2016) found that students with earlier access to on-demand hints did not perform better than those with only later access to on-demand hints, and, in fact, these students took significantly more time to complete the activity.

There are a number of possible reasons for these findings, including contextual factors specific to summer activities (i.e., distant deadlines requiring students to self-regulate their learning patterns). However, these findings raise questions about the effectiveness of providing students with hints in the early problems of an online assignment. In this study, we attempted to replicate these findings by applying the same experimental methodology to a different sample of students, answering the same problems during the regular academic year in order to determine if the findings from the initial study generalized to a new context.

## Research questions

The goal of the present study was to determine how well the findings of the initial study (Inventado et al. 2016) generalized to other learning contexts. The same experimental methodology in the initial study was used, but it was applied on data collected from a sample of students who answered math problems during the regular academic year (from January to May 2016). The following discussions use the terms *summer* study to refer to the initial study conducted by Inventado et al. (2016), and *academic year* study to refer to the replication study. This paper aimed to answer the following research questions:

- *Research question 1*: *Do results observed in the summer study replicate those observed in the academic year study?* The academic year sample of students were exposed to the same experimental methodology as the summer sample. Replication would involve students assigned to receive hints within the first three problems of their assignment to take significantly longer to complete their work without any significant performance improvement. Other outcome measures tested in the summer study were also used to evaluate students’ learning behavior, which are discussed in Tables 1 and 2 of the next subsection.

**Table 1:** Performance measures

Measure	Summary
Mastery speed	The total number of problems completed before students achieve mastery (Xiong et al. 2013; Xiong et al. 2014). In the ASSISTments’ Skill Builder assignment, mastery is defined as accurately answering three consecutive problems. When a student achieves mastery, they are no longer assigned more problems in that Skill Builder. In the current study, students used anywhere from 3–40 problems to master the Skill Builder, with an average of 8 problems.
Percent correct	The number of problems answered correctly out of the total number of problems attempted. Compared to the mastery speed measure, percent correct is more susceptible to guessing and provides less penalty to slipping because correct answers are credited even though prior or future problems are answered incorrectly. Baker et al. (2008) define guessing as providing the correct answer despite not knowing the skill and slipping as providing an incorrect answer despite knowing the skill.
Total answer-attempts	The total number of answer attempts students made while solving problems in the Skill Builder. Take note that this is different from the number of problems answered in the mastery speed measure. Low total answer-attempt counts may indicate that the student has sufficient knowledge to answer problems, while high answer-attempt counts may indicate difficulty with the problem or possibly engagement in systematic guessing or gaming behavior (Baker et al. 2004).
Total regular-hint count	The total number of hints requested by students throughout the Skill Builder that did not reveal the correct answer (i.e., non-bottom-out hint).
Total bottom-out-hint count	The total number of bottom-out hints requested by students while solving the Skill Builder.
Problems with hints	The number of problems in the Skill Builder in which the student requested either regular or bottom-out hints.
Attrition	The case when a student failed to complete the problem set, or effectively “dropping out” of the Skill Builder.

**Table 2:** Temporal measures

Measure	Summary
Completion time (minutes)	The amount of time it took students to complete the Skill Builder. It was computed by subtracting the timestamp when a student started solving the first problem from the timestamp when a student completed the last problem in the Skill Builder.
Total time-on-problem (minutes)	The total time a student spent on each problem until the Skill Builder was completed. Specifically, time-on-problem was first computed for each problem solved by subtracting the timestamp when a student started to answer the problem from the timestamp when a student completed the problem (i.e., a correct answer to the problem is given by the student) then summed across problems to get the total time-on-problem.
Total time-between-problems (minutes)	The total time students spent outside answering problems in ASSISTments before completing the Skill Builder. Specifically, time-between-problem was first computed by subtracting the timestamp when the student completed the prior problem from the timestamp when the student started the next problem then summed to get the total time-between-problems.
Winsorized total time-on-problem (minutes)	The sum of time-on-problem values that were winsorized using a 10% cutoff.
- *Research question 2*: *Were there individual differences between the student samples in the summer study and that in the academic year study?* Similarities and differences between student samples may influence the replicability of findings. Table 3 in the next subsection lists the individual-difference measures considered.

**Table 3:** Individual difference measures

Feature	Summary
Gender	ASSISTments does not ask users to self-report gender. Gender is determined by comparing a user’s first name to a global name database from which gender is inferred. Gender may be set to “Unknown” if a user’s name is not found in the database.
Grade level	The student’s grade level. Although the Skill Builder used in the experimental methodology was designed for grade 8 students, any grade level teacher could freely assign the Skill Builder. For example, a teacher might have assigned it to challenge advanced students in lower grade levels, to provide practice for grade 8 students, or to review the relevant prior knowledge of students in higher grade levels.
Prior Skill Builder count	The number of prior ASSISTments Skill Builders a student has started to answer.
Prior Skill Builder completion percentage	The number of prior ASSISTments Skill Builders that a student completed out of all the Skill Builders he/she started.
Prior problem count	The number of prior individual ASSISTments problems that a student has answered.
Prior problem correctness percentage	The percentage of ASSISTments problems that a student has answered correctly out of all problems he/she answered, prior to participation in the studies.
- *Research question 3*: *Were there differences in external factors between the studies?* Aside from the students’ individual differences, differences in the learning settings might have also influenced the findings. Table 4 in the next subsection lists the external factors that were considered.

**Table 4:** External factors

Feature	Summary
School location	The number of students in the experiment who were enrolled in schools located in either urban, suburban, or rural areas.
Assignment duration (weeks)	The time allotted by the teacher for students to complete the Skill Builder.

## Methods

This section first discusses the ASSISTments online learning system that was used for conducting both studies, followed by the randomized controlled trial (RCT) methodology used, as well as descriptions of the various outcome measures used to evaluate differences between conditions.

### ASSISTments

**Fig. 1:**
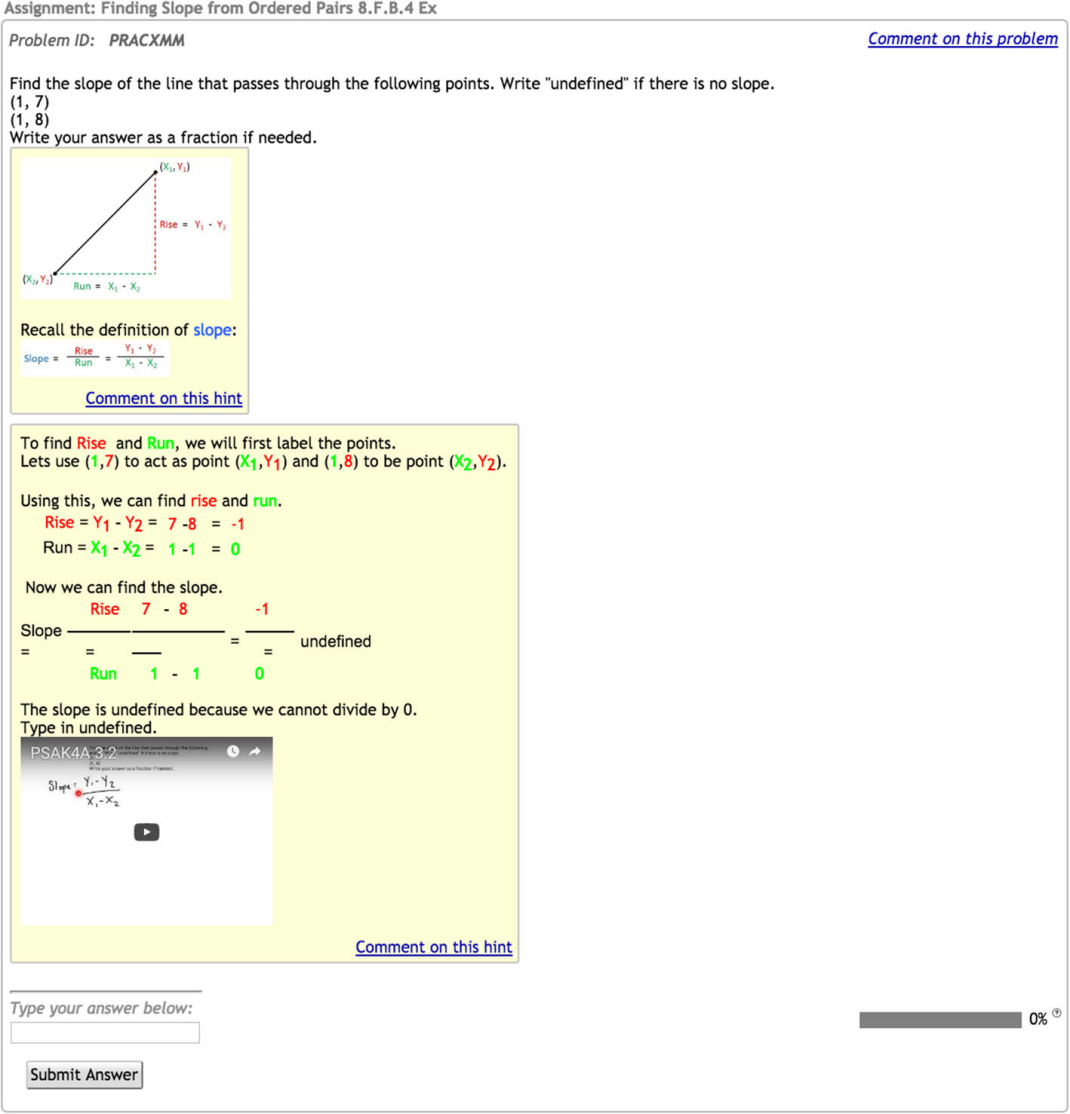
No Hints-early screenshot. An example question from the no-hints-early condition, showing a bottom-out hint

**Fig. 2:**
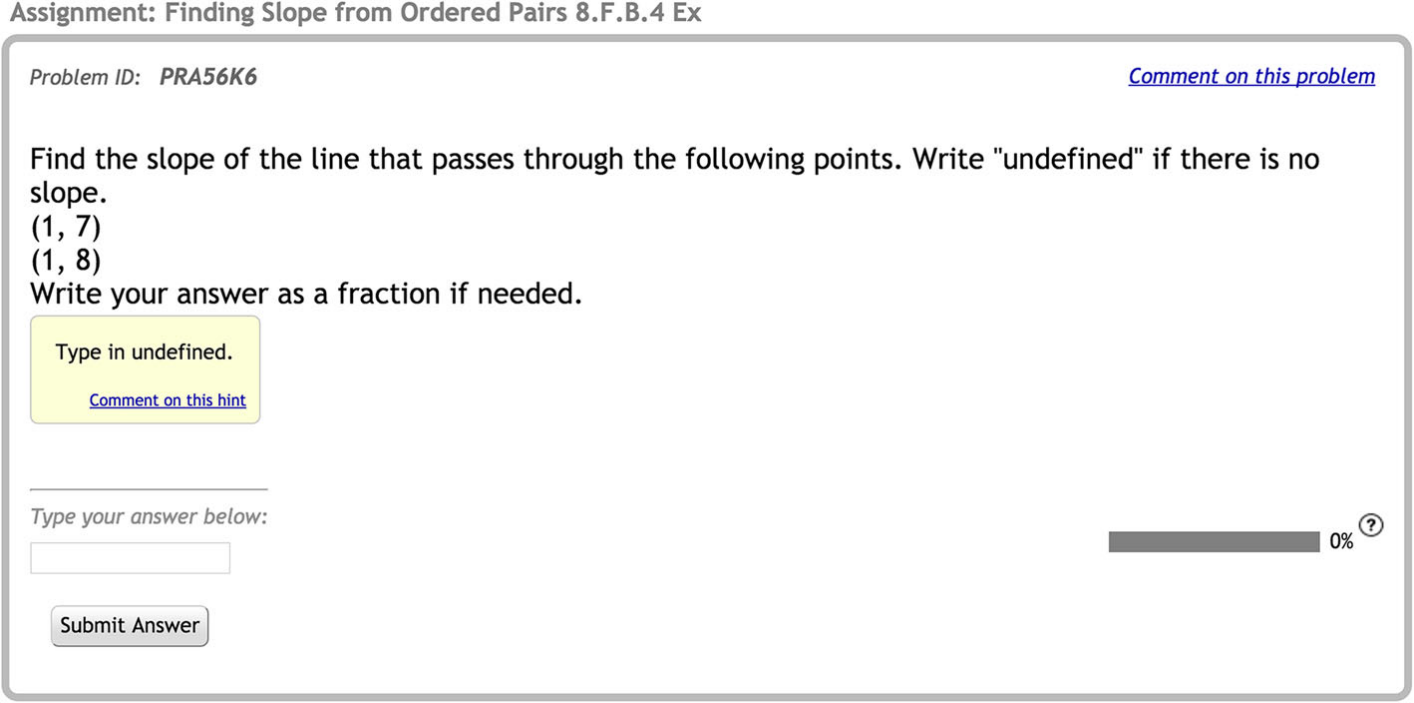
Hints-early screenshot. An example question from the hints-early condition, showing associated hints

As in Inventado et al. (2016), the RCTs in this study were implemented in the ASSISTments online learning system (Heffernan and Heffernan 2014). The system was designed primarily for middle school mathematics and allows teachers to easily create and assign their own problem sets (including questions, associated solutions, mistake messages, and feedback) or to select curated sets from the ASSISTments Certified Library (assignments vetted by ASSISTments’ expert team) (Heffernan and Heffernan 2014; Razzaq and Heffernan 2009). General skill-based problem content, book work linked to many of the top mathematics texts in the nation, and mastery-learning-based “Skill Builders” all offer learning experiences that simultaneously support student learning and provide automated formative assessment through real-time data to teachers (Heffernan and Heffernan 2014). Figures 1 and 2 show screenshots of the types of ASSISTments problems used in the present work. ASSISTments is also evolving into a platform that enables researchers to conduct RCTs and retrieve reports for efficient data analysis within an authentic educational environment (Heffernan and Heffernan 2014; Ostrow et al. 2016). The system logs learning-related features at multiple granularities (e.g., problem text, problem type, student actions, and timestamps) and provides researchers with a number of useful student, class, and school-level covariates.

Within Skill Builders, the type of assignment considered in the present work, students solve problems randomly selected from a skill bank until they are able to reach “mastery” by accurately solving a predefined number of problems in a row (the default is three). While ASSISTments recently added the capability for students to earn partial credit while working through these types of problems, mastery can only be achieved by solving the consecutive problems accurately on the first attempt. This means that students can ask for assistance from the system, but that the problem will be marked incorrect. This is an important design behavior to consider in the context of the present work because hint feedback is the topic of interest.

### RCT methodology

**Fig. 3:**
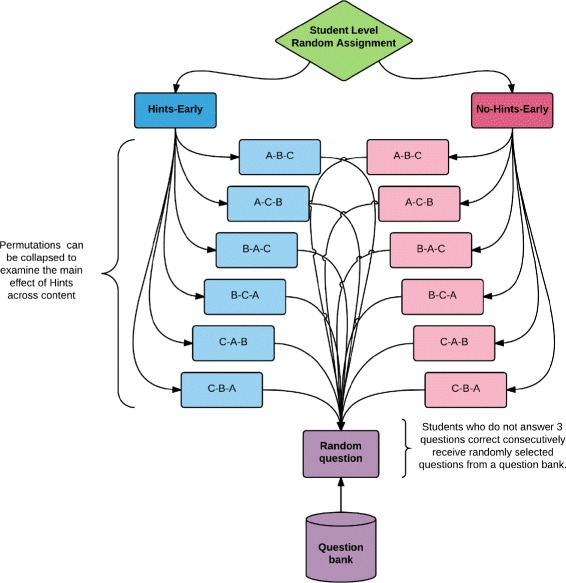
RCT methodology. Research methodology depicted as a flow chart (Inventado et al. 2016)

The RCT used in the academic year study was identical to the summer study conducted by Inventado et al. (2016) that assigned students to one of two conditions designed to provide different options for student behaviors in the first three problems of the Skill Builder: no-hints-early (NHE) and hints early (HE). This methodology is illustrated in Fig. 3. Students assigned to the NHE condition were only allowed to request bottom-out hints (a hint that provides the correct answer) in the first three problems, but were allowed to request on-demand-hints for all remaining problems. On-demand hints provide students with a progressively more detailed sequence of hints upon each hint request. As students must eventually enter the correct answer to move on to the next problem in the system, the last hint in the sequence provides the correct answer, termed the bottom-out hint. This practice keeps students from getting stuck within their assignments. In contrast, students assigned to the HE condition were allowed to request on-demand hints throughout the full assignment. All students, regardless of condition, received correctness feedback when they submitted answers. An example of correctness feedback is, “Sorry try again: ‘2’ is not correct.” Figures 1 and 2 demonstrate the differences by condition. In the NHE condition, students were only offered a “Show Answer” button in the lower right corner of the screen for the first three problems, allowing those who were stuck to move on to the next problem and eventually complete the Skill Builder (a design seen in early intelligent tutors (Schofield 1995)). The HE condition allowed students to access on-demand hints at any time by clicking on a button in the lower right corner of their screen labeled “Show hint *x* of *y*.” The problem remained on the screen while video tutorials and text-based hints were simultaneously delivered (although redundant, text-based hints ensured access when school firewalls or connectivity issues may have limited access to YouTube). The system marked the students requesting hints, or ultimately the answer, as incorrect. While this may be a deterrent to hint usage, the idea of penalizing assistance may actually speak to self-regulated learning in a mastery-learning environment, as students requesting and learning from feedback early on may be more likely to accurately solve the next three consecutive problems.

The problems used in the present work were chosen from ASSISTments Certified content designed to address the eighth grade Common Core State Standard “Finding Slope from Ordered Pairs” (National Governors Association Center for Best Practices, Council of Chief State School Officers 2010). Within each condition, the first three problems containing the experimental manipulation (HE vs. NHE) were randomly ordered to minimize the potential for peer-to-peer cheating (i.e., students may have received the first three problems in the order A-B-C, A-C-B, B-A-C, etc., as depicted in Fig. 3). Following these first three problems, students requiring content to master the skill were provided problems from the original ASSISTments Certified Skill Builder for the given topic. That is, all students were exposed to a set of validated questions randomly sourced from previously certified content built and delivered by the WPI ASSISTments team. These remaining problems were not considered part of the experimental manipulation and followed a traditional Skill Builder format requiring students to accurately answer three consecutive problems to reach mastery. In order to provide all students with adequate learning support, all students were permitted on-demand hints upon reaching these additional problems.

### Measures and features used for analyses

Several measures were used to investigate differences between conditions and student populations in both studies. First, Table 1 lists seven performance measures that described students’ progression toward completing the assigned Skill Builder. Second, Table 2 lists four temporal measures that described the time students spent performing on- and off-task activities while solving problems within the Skill Builder. Third, Table 3 lists six measures that described students’ individual differences. Finally, Table 4 lists two measures that described the students’ learning context.

A limitation of ASSISTments’ current logging mechanism is its susceptibility to conditions that may influence temporal measures such as disconnection from the network, shifts between learning activities, off-task behavior, submitting assignments past the deadline, and others. The time-on-problem measure, for example, had values as long as 4 months in the data logged for the academic year study. Time-on-problem is the amount of time a student spends answering a single problem. Obviously, these values were outliers possibly reflecting students that opened a problem and walked away for extenuous periods of time. To remedy this issue, we applied winsorization, a technique that truncates values of elements in a set that are either above the upper limit or below the lower limit (Ghosh and Vogt 2012). For example, we applied a 10% upper and lower bound cutoff on the time-on-problem feature of the academic year sample population, leaving 80% of the raw data. Winsorization produced a range between 11 and 404 s (inclusive) so any value below 11 s was changed to 11 s, and any value above 404 s was changed to 404 s. Upper and lower bound limits in winsorization are often set to 5%, but applying it to the academic year sample resulted in still unrealistic values. Specifically, a 5% cutoff resulted in an upper bound of 10,920 s for the time-on-problem measure, suggesting that students were taking over 3 h to solve a single problem.

The winsorization process transformed the time-on-problem values to filter outliers. However, this altered the time to the next problem, which complicated the definition of time-between-problems. Therefore, we opted to use a clearer definition of time-between-problems that considered actual time duration values without winsorization.

## Results

The RCT methodology was implemented in ASSISTments and has been running since May 2015. Data used in the analyses for the summer study was collected from students who received the Skill Builder as part of their summer math activity, coupled with numerous other assignments to be completed between June and September 2015 (Inventado et al. 2016). The academic year study investigated new data from the same RCT, focusing on data collected from a different sample of students between January 2016 and May 2016. The following subsections compare the results between the academic year study and the summer study. The values of the outcome measures used in the analyses were mostly skewed, so non-parametric tests were applied.

### Research question 1

A chi-squared test was first conducted to check for differences in attrition according to condition assignment, where attrition is defined as a student’s failure to complete the Skill Builder, or effectively “dropping out” of the assignment. As observed in Inventado et al. (2016) with the summer sample, there were no significant differences in attrition by condition in the academic year sample population. Condition distributions were also well matched for gender, grade level, school location, and prior performance measures such as prior Skill Builder count, prior Skill Builder completion, prior problem count, and prior problem correctness. Multiple Mann-Whitney *U* tests were conducted to find differences on the seven performance and four temporal measures by condition. Table 5 shows the results from the Mann-Whitney *U* tests conducted on the outcome measures in both the summer study and the academic year study. Benjamini-Hochberg post hoc corrections were performed to account for multiple testing. Table 5 shows the median values for each condition and the corresponding effect sizes calculated using Cliff’s delta (Cliff 1996).

**Table 5:** Medians and effect sizes of performance- and temporal-based dependent variables by condition for both samples

Dependent variables	Summer sample (*N*=390)			Academic year sample (*N*=289)		
	HE (*N*=201)	NHE (*N*=189)	Cliff’s *d*	HE (*N*=141)	NHE (*N*=148)	Cliff’s *d*
Mastery speed	8.00	9.00	.04	8.00	8.00	.04
Percent correct	0.64	0.60	.01	0.67 ^∗∗^	0.59 ^∗∗^	.14
Total answer-attempts	14.00	15.00	.00	14.00	15.00	.06
Total regular-hint count	0.00 ^∗∗^	0.00 ^∗∗^	.27	0.00 ^∗^	0.00 ^∗^	.11
Total bottom-out-hint count	0.00 ^*†*^	0.00 ^*†*^	.10	0.00 ^∗∗^	1.00 ^∗∗^	.24
Problems with hints	0.00	0.00	.03	0.00 ^∗∗^	1.00 ^∗∗^	.18
Completion time	44.90 ^∗^	20.77 ^∗^	.01	21.12	17.88	.19
Total time-on-problem	24.12	18.80	.08	14.73	13.05	.04
Total time-between-problems	0.27	0.27	.07	0.27	0.23	.01
Winsorized total time-on-problem	16.32	15.40	.00	11.64	11.86	.00

*Note:* Value ranges differ across variables; *HE* hints early condition; *NHE* no-hints-early condition; ^*†*^*p* < 0.1, ^∗^*p* <.05, ^∗∗^<.01

Analyses on the academic year sample did not reveal any differences in mastery speed or answer attempts between conditions, but showed that students in the HE condition answered significantly more problems correctly compared to students in the NHE condition. Additionally, students in the HE condition were more likely to request hints with the HE group asking for an average of 1.22 hints, while the NHE group only asking for an average of 0.84 hints. In contrast, students in the NHE condition were more likely to request bottom-out hints and also more likely to request either regular or bottom-out hints in the problems they solved.

Students in the HE condition for both samples had access to hints on the first three problems while students in the NHE condition only had access to bottom-out hints. Due to this constraint, students in the HE condition were inherently more likely to request regular hints, so we first analyzed students’ help-seeking behavior on the first three problems and subsequently on any remaining problems required to complete the Skill Builder, as shown in Table 6. Note that fewer students answered remaining problems (more than three problems) due to either Skill Builder completion or attrition. As expected based on the experimental design, students in the HE condition requested significantly more hints on the first three problems: an average of 0.55 hints, compared to no hint requests by students in the NHE condition (as it was not possible). Students in the NHE condition requested significantly more bottom-out hints (answers), suggesting that when provided a hint series, students in the HE condition may have learned from early hints in the progressive sequence, frequently entering the correct answer without requiring the bottom-out hint. Finally, students in the NHE group were more likely to request help when they answered remaining problems. There did not seem to be any strong differences between conditions for the remaining problems, although a trend was observed in which students in the NHE condition requested help more frequently on problems (*M* = 0.52, *SD* = 1.12) than students in the HE condition (*M* = 0.37, *SD* = 1.32).

**Table 6:** Medians and effect sizes of help-seeking-behavior measures by condition for both samples

Dependent variables	Summer sample (*N*=390)			Academic year sample (*N*=289)		
	HE	NHE	Cliff’s d	HE	NHE	Cliff’s d
First three problems only	*N* = 201	*N* = 189		*N* = 141	*N* = 148	
Total regular-hint count	0.00 ^∗∗^	0.00 ^∗∗^	.48	0.00 ^∗∗^	0.00 ^∗∗^	.37
Total bottom-out-hint count	0.00	0.00	.13	0.00 ^∗∗^	10.00 ^∗∗^	.27
Problems with hints	0.00	0.00	.03	0.00 ^∗∗^	10.00 ^∗∗^	.19
Remaining problems only	*N* = 128	*N* = 123		*N* = 80	*N* = 91	
Total regular-hint count	0.00	0.00	.38	0.00	0.00	.49
Total bottom-out-hint count	0.00	0.00	.37	0.00	0.00	.42
Problems with hints	0.00	0.00	.39	0.00 ^*†*^	0.00 ^*†*^	.13

*Note:* Fewer students answered remaining problems due to Skill Builder completion or attrition; HE - hints early condition; NHE - no-hints-early condition; ^*†*^p<0.1, ^∗^p<.05, ^∗∗^p<.01

Results of the analyses in the academic year sample showed no differences in the amount of time it took students to complete the Skill Builder, which was not consistent with the findings in the summer study. Moreover, there were no differences in student attrition between conditions within each sample, but a chi-squared test showed that students in the academic year sample were significantly less likely to complete the Skill Builder (74% completion) compared to students in the summer sample (84% completion), *X*^2^(1, *N* = 679) = 9.17, *p* <.005. Performance and temporal measures could not be compared across sample populations because of the differences in attrition. Students in the academic year and summer samples were randomly assigned to each condition (i.e., HE and NHE), which led us to believe that both samples were internally valid and that other factors might have influenced differences in attrition.

### Research question 2

Differences in the findings on condition assignment and students’ likelihood to complete the Skill Builder led to the investigation of other contextual factors such as individual differences among students. Several tests were conducted to compare individual differences between the summer and academic year sample population. The tests compared both sample populations and did not consider random assignment to early or late hint access. First, chi-squared tests were conducted to find differences in the distribution of gender between both sample populations, which showed no significant differences. However, a chi-squared test revealed significant differences in the distribution of grade level between the sample populations, *X*^2^(8, *N* = 679) = 581.37, *p* <.0001. Students in the summer sample were in either the ninth or the tenth grade while students in the academic year sample reported greater variation in grade levels, as presented in Table 7.

**Table 7:** Distribution of student gender and grade level in the summer and academic year sample populations

Feature	Summer sample	Academic year sample
Gender	Female (188), Male (159), Unknown (43)	Female (132), Male (116)
Grade level	Gr. 9 (295), Gr. 10 (95)	Gr. 6 (12), Gr. 7 (15), Gr. 8 (170), Gr. 9 (9), Gr. 10 (18), Gr. 11 (23), Gr. 12 (24), Unspecified (18)

Several Mann-Whitney *U* tests were conducted to find differences in prior performance measures between the two sample populations, and again, Benjamini-Hochberg post hoc corrections were applied to control for multiple testing. Table 8 shows the results of the tests conducted where significant differences were found in the number of Skill Builders answered, the percentage of Skill Builders completed, and the number of problems answered between the summer and academic year samples. However, there were no significant differences in the percentage of problems that the two sample populations answered correctly. Table 8 also presents the corresponding median and Cliff’s delta values for each of the tests conducted.

**Table 8:** Medians and effect sizes of prior-performance-based dependent variables for both sample populations

Dependent variable	Summer sample	Academic year sample	Cliff’s *d*
Prior Skill Builder count	19.00 ^∗∗^	24.00 ^∗∗^	.25
Prior Skill Builder completion percentage	1.00 ^∗∗^	0.93 ^∗∗^	.25
Prior Problem count	148.00 ^∗∗^	706.00 ^∗∗^	.71
Prior Problem correctness percentage	0.72	0.71	.07

*Note:* ^∗∗^*p*<.01

### Research question 3

Features in the data that described external factors were limited but were shown to be significantly different between the two samples. First, all students in the summer sample were enrolled in suburban schools (*N* = 390) while students in the academic year sample were enrolled in suburban (*N* = 246), urban (*N* = 23), rural (*N* = 15), and an unidentified location (*N* = 31) schools, *X*^2^(3, *N* = 679) = 103.65, *p* <.0001.

Second, the time allotted to complete the Skill Builders in the summer sample was significantly longer (*Mdn*=13 weeks) than the academic year sample (*Mdn*=1.2 weeks), *U*(0), *p*<.0001. On the one hand, the long time allotted for students in the summer sample was consistent with the purpose of the Skill Builder, which was a part of a summer math activity. On the other hand, the time allotted for students in the academic year sample was consistent with the usual time allotted for homework during the regular school year.

### Working model

**Fig. 4:**
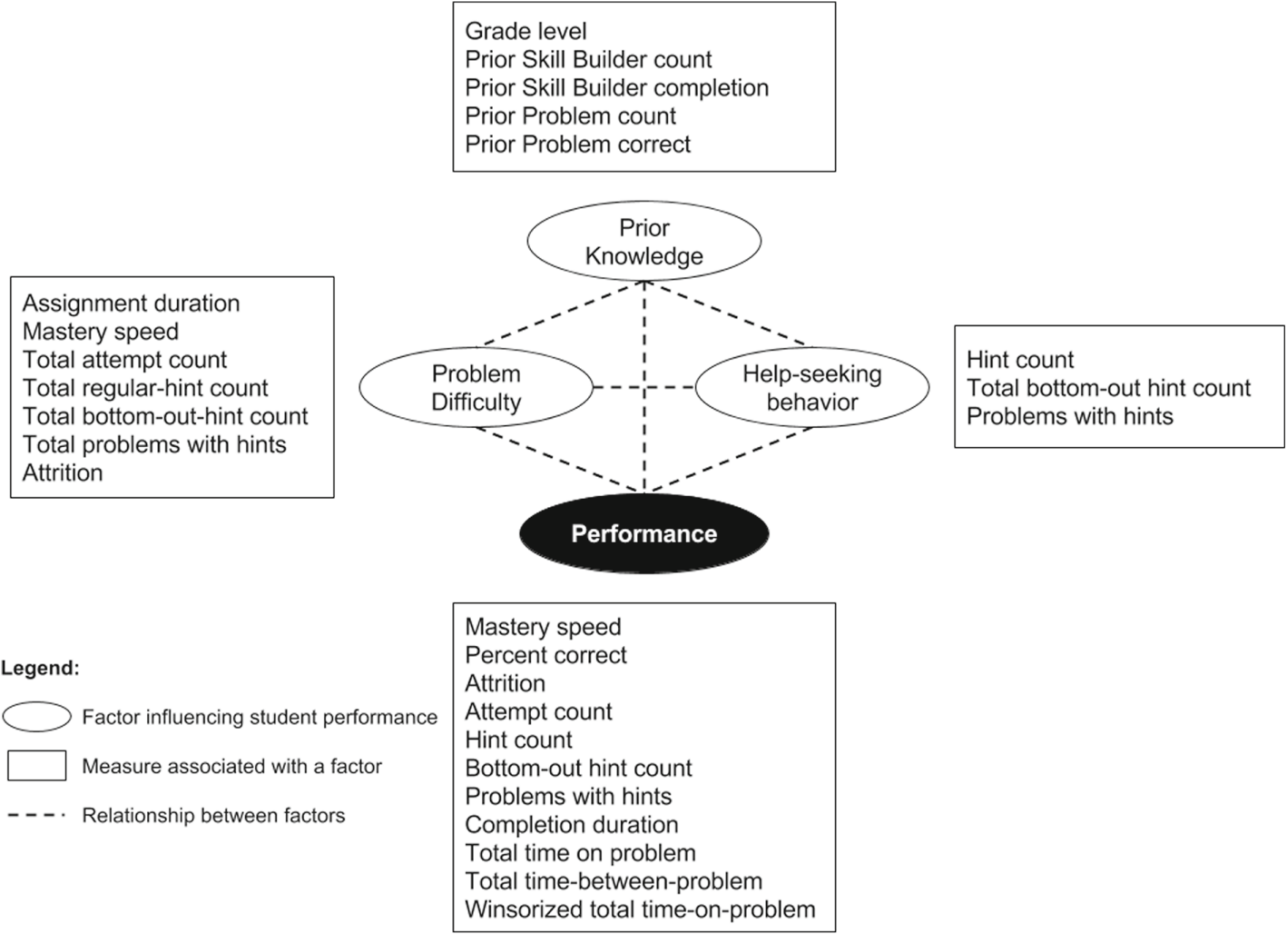
Working model used to explain differences between sample populations

We attempt to explain differences in findings between the summer and academic year studies in Fig. 4. The model consists of three factors that we think influenced learning performance (represented by circles) and sets of measures collected from the data (represented by boxes) to describe these factors. The three factors we considered are prior knowledge, problem difficulty, and help-seeking behavior. Our working model was based on the data collected from both of our sample populations and the measures available from ASSISTments.

The first factor is prior knowledge, or the prerequisite understanding necessary for students to solve a problem. Grade level may be a good measure for prior knowledge wherein students from higher grade levels are assumed to have the knowledge required to solve problems intended for lower grade levels. For example, students in grade nine should have already acquired the skills needed to solve problems intended for grade eight. Prior Skill Builder count and prior Skill Builder completion percentage describe how many problems a student has already completed in ASSISTments providing an idea of a student’s learning history. However, not all of their previously solved problems are necessarily related to the current problem. Similarly, prior problem count and prior problem correctness offer insight regarding how well the student mastered prior skills without a standardized connection to the current content.

The second factor is problem difficulty. Problems that deal with complex topics are inherently difficult. However, low-quality instructional designs can potentially increase problem difficulty. Short assignment deadlines can contribute to problem difficulty as students, especially those with low prior knowledge, who may fail to complete the activity on time. ASSISTments measures that may gauge problem difficulty include mastery speed and attempt count (how many problems and attempts it takes students to master a skill), hint requests (how much help students need before they are able to solve a problem), and attrition (students’ persistence in solving the problem).

The third factor is help-seeking behavior, which describes cases when students request help to solve a problem that is inherently difficult or to acquire the knowledge needed to solve it. Help-seeking behavior can be measured by the frequency of hint requests, bottom-out hint requests, and the problems with hints measure. Requesting regular hints alone compared to requesting a bottom-out-hint may indicate higher levels of self-efficacy because a student could request just enough help to solve the rest of the problem on his/her own. Recent studies, however, show that students who need assistance are often the ones who either do not request help or request help but use it ineffectively (Baker et al. 2004; Puustinen 1998; Ryan et al. 1998).

Student performance can be measured by mastery speed, percent correct, and attrition, which capture how quickly or accurately a student solves the problem and when a student decides to quit. Low attempt counts and the lack of regular and/or bottom-out-hint requests may also indicate strong performance because students can easily identify the answer without help. Short completion time, time-on-problem, and time-between problem measures may indicate that a student knows how to solve the problem and is motivated to complete it.

Students’ prior knowledge, the difficulty of a problem, and help-seeking behavior help determine their performance in solving a problem. Students who acquired relevant prior knowledge are likely to perform well. Students who lack prior knowledge may struggle to solve difficult problems but can succeed with the help of appropriate learning support (Clark and Mayer 2016; Hume et al. 1996; Vygotsky 1962).

## Discussion

Despite applying the same experimental methodology, results from the summer and academic year study were different. This finding potentially speaks to a number of interesting issues including the replication crisis that is becoming apparent in many fields, assumptions regarding the cherry picking of results and the “file drawer problem,” and the complex nature of conducting at-scale RCTs using online learning technologies. Recent research has revealed that replication is difficult to achieve and that many of the results published in fields like medicine and psychology may in fact highlight false positives or findings that fail to generalize (Ioannidis 2015). Even when sound science is applied, following strict methodologies can still lead to the reporting of significant findings without attempt at generalization. Perhaps even more dangerous, findings that fail to show significance are typically lost to the world, commonly labeled the “file drawer problem,” or when only significant findings lead to publication. The present work depicted a scenario in which the summer study findings looked unimpressive, while the academic year study findings aligned to common hypotheses in the field. If this pattern had been reversed, philosophical questions regarding generalizability would not need mentioning, and results simply would have added evidence to the field. This issue raises questions regarding the realm of research conducted through online learning systems that can be used for quick replication, tackling issues longitudinally and across highly heterogeneous samples. As educational research evolves within technology’s grasp, it is critical to understand how data quantity, quality, and heterogeneity interact. ASSISTments is currently a rather unique platform for research, allowing researchers from around the world to conduct RCTs within authentic learning environments that can effectively be sampled across time. This methodology will likely grow in popularity as other systems advance to allow similar flexibility in research, and thus, it is necessary to begin considering the ramifications of these in vivo approaches.

In the present work, we used the aforementioned working model to analyze the results of both studies and provide possible interpretations of why their results differed. Students from the summer sample population appeared to have more prior knowledge than students in the academic year sample. On the one hand, students from the summer sample population were mostly grades nine and ten students, who should have already acquired the skills needed to master the Skill Builder (intended for eighth grade students) or possibly even worked on the same Skill Builder in a previous year. On the other hand, the majority of the students in the academic year sample population were between grades six and eight. Although they answered more problems and Skill Builders in ASSISTments, it is likely that these students answered problems intended for earlier grade levels that were not specific to the current skill. Further research may be needed to better understand the impact of prior ASSISTments experience on performance and hint usage.

Students from the summer sample population might have found the problem easier to solve compared to students in the academic year sample population. First, students in the summer sample probably had more sufficient prior knowledge as described in the previous discussion. Second, the Skill Builder would have been easier for students in the summer sample population because they had around 3 months to complete the Skill Builder compared to students in the academic year sample who only had around 1 week to complete it. Third, although there were no differences in attrition between conditions for the individual studies, there were significantly more students in the academic year sample population who did not complete the Skill Builder compared to those in the summer sample population. More students in the academic year sample population may have struggled with the Skill Builder, which led them not to complete it. Moreover, the problems may have been more difficult for students in the NHE condition of the academic year sample population, causing them to ask for hints and answers in more problems compared to students in the HE condition. This may have suggested that students in the HE condition were learning from their early hint usage, while those in the NHE condition were not as well assisted by the bottom-out hints and therefore continued to need additional assistance in later problems. Differences in this kind of behavior between conditions were not seen in the summer sample population.

There were more significant differences in help-seeking behavior between conditions in the academic year sample population compared to those in the summer sample population. Students in the academic year sample population may have had less prior knowledge than students in the summer sample population, which led them to ask for more help to complete the Skill Builder. Students in the HE condition were naturally more likely to ask for regular hints because they had early access to hints. However, students in the NHE condition asked for significantly more bottom-out hints than students in the HE condition who could also see bottom-out hints after asking for all available regular hints. This implies that students in the HE condition may have learned from their early hint access, requiring fewer hints and answers later.

Finally, students in the HE condition of the academic year sample performed significantly better than students in the NHE condition, which was not observed in students within the summer sample population. Students in the HE condition of the academic year sample population had a significantly higher percentage of problems answered correctly than students in the NHE condition. As discussed previously, students in the academic year sample population were likely to have lower prior knowledge, and to experience more difficulty answering the Skill Builder due to shorter assignment deadlines. In this case, students in the HE condition had earlier access to hints, which likely helped them to perform better. This finding is consistent with literature describing the effectiveness of explanatory feedback in helping students learn skills they would be otherwise unable to achieve on their own (Clark and Mayer 2016; Hume et al. 1996; Kleij *et al*^2015^*; Vygotsky*^1962^). In contrast, students from the summer sample population who were exposed to the HE condition might have previously acquired the skills to solve the problem and just used the hints for review, which might have had similar effects with students in the NHE condition who only received bottom-out-hints early in the problem set. Students assigned to the HE condition in the summer sample population took significantly more time to complete the Skill Builder but spent roughly the same amount of time solving individual problems compared to students assigned to the NHE condition. Some possible interpretations discussed in Inventado et al. (2016) were that students in the HE condition spent more learning time outside of ASSISTments, possibly reviewing or relearning concepts described in the hint, locating and answering easier Skill Builders that were also assigned in their summer math activity, or putting off their work because of perceived difficulty, lack of self-efficacy, or apathy. In the case of the academic year sample population, students were expected to complete the Skill Builder within a week, which may have encouraged students to complete their assignment (or dropout) faster.

## Conclusions

Hints, or any help strategy for that matter, may be effective in particular situations, but there are other factors in play that may decrease their effectiveness. In this paper, two instantiations of the same experimental methodology applied to two different student sample populations were compared. Comparison of the summer and academic year studies revealed that students in the academic year sample population who were given earlier access to on-demand hints had a significantly higher answer correctness percentage. In the summer sample population, however, students given earlier access to on-demand hints did not perform any better than students who were only given access to correctness feedback when they started answering the Skill Builder. They also took more time to complete the Skill Builder.

Three factors were used to investigate differences between the two sample populations that may explain why findings in the summer study did not replicate to the academic year study: prior knowledge, problem difficulty, and students’ help-seeking behavior. Measures from the data collected were used to describe these factors. On the one hand, students from the academic year sample population appeared to have less prior knowledge, experienced more difficulty answering problems, and sought more help, wherein earlier access to hints helped them perform better than students with later access to hints. On the other hand, students from the summer sample population appeared to have more prior knowledge, experienced less difficulty answering problems, and sought less help, wherein earlier access to hints was not any better than later access to hints.

The results of these studies indicate that it is not enough to simply base instructional design decisions on prior research. It is also important to validate the effectiveness of a design especially when there are contextual differences between the learning settings and student populations for whom a prior design was applied. This is increasingly important for online learning systems like ASSISTments that cater to thousands of learners from diverse backgrounds, making it difficult to develop optimized instructional designs. It becomes important to not only identify good instructional designs but to also identify which students may benefit most from the design and the type of learning settings in which various designs are most effective. Generalization research will be the key as similar platforms advance in the capacity for in vivo research.

This work has several limitations including the differential attrition rate between the two sample populations, which made it unreliable to perform in-depth analyses across populations. Take note that differential attrition was not a problem for the individual studies and each study was internally valid as students were randomly assigned to conditions. The experimental methodology was only applied to two sample populations, but analyses of more varied populations are needed to develop more robust instructional designs. Hierarchical analyses could also be used to check for interactions between learning measures, as well as account for the potential variance in school-level variables; however, larger samples would likely be required to perform such analyses reliably. We plan to address these limitations in a future work in order to uncover more intuition as to how hints may be properly utilized in different domains and learning scenarios.

## Abbreviations

HE: Hints early
NHE: No-hints early
RCT: Randomized controlled trial
